# Utilization of public health care by people with private health insurance: a systematic review and meta-analysis

**DOI:** 10.1186/s12889-020-08861-9

**Published:** 2020-07-23

**Authors:** Congcong Zhang, Chenwei Fu, Yimin Song, Rong Feng, Xinjuan Wu, Yongning Li

**Affiliations:** 1 Department of International Medical Servicers, Peking Union Medical College Hospital, No.1 Shuaifuyuan, Wangfujing Dongcheng District, Beijing, 100730 China; 2 Department of Nursing, Peking Union Medical College Hospital, No.1 Shuaifuyuan, Wangfujing Dongcheng District, Beijing, 100730 China

**Keywords:** Private health insurance, Health care utilization, Systematic review, Meta-analysis

## Abstract

**Background:**

The objective of this systematic review was to explore the association between private health insurance and health care utilization.

**Methods:**

We searched the MEDLINE, EMBASE, and Cochrane Central Register of Controlled Trials (CENTRAL) electronic databases for relevant articles since 2010. Studies were eligible if they described original empirical research on the utilization of public health care by individuals with private health insurance, compared with individuals without private insurance. A pooled measure of association between insurance status with health care utilization was assessed through meta-analysis.

**Results:**

Twenty-six articles were included in the final analysis. We found that patients with private insurance did not use more public health care than people without private insurance (*P* < 0.05). According to the subgroup analysis, people with private insurance were more likely to be hospitalized than people with no insurance (OR 1.67; 95% CI, 1.18 to 2.36).

**Conclusions:**

People with private insurance did not increase their use of health care (outpatient services), compared to those without private insurance. Private health insurance coverage may ease the financial burden on patients and on the public health insurance system.

## Background

As most countries across the world face rapidly escalating health expenditures, exorbitant out-of-pocket payments have resulted in high demand for supplementary private health insurance [1, 2]. For instance, in 2015, approximately 80% of households in the United States had to purchase at least one private health insurance plan, and more than 25% of Brazilians had private health insurance in 2019 [3, 4].

The role of private health insurance is fiercely debated. Some researchers believe that the use of private health insurance should be encouraged in order to ease the financial burden on patients and on social healthcare systems [5]. However, others maintain that the use of private health insurance will contribute to the current rapid increase in health expenditures, induce fragmentation of the healthcare system, and aggravate social inequity by increasing the gap in health care utilization between opposite ends of the socioeconomic spectrum [6, 7]. One of the critical controversies over private health insurance is its potential impact on health care utilization. If individuals with private health insurance increase their utilization of health care, the result will be inequity in health care utilization between those who purchase private health insurance and those who do not.

Although previous studies have examined the effect of private insurance on the utilization of public health care in specific countries [3, 6], no study published to date has systematically investigated the issue on a global scale. However, it is necessary for stakeholders to understand the role that private insurance companies play the use of healthcare services from a macro perspective. The objective of this systematic review was to synthesize available evidence to compare the effect of private health insurance to the effect of having no (private) insurance or public health care insurance on the utilization of health care (inpatient and outpatient resources) among all kinds of patients worldwide.

## Methods

### Search strategy

This systematic review was performed in accordance with the recommendations of the Preferred Reporting Items for Systematic Reviews and Meta-Analyses (PRISMA) guidelines, but the review protocol was not registered [8]. Two reviewers searched MEDLINE, EMBASE, and the Cochrane Central Register of Controlled Trials (CENTRAL) databases for relevant articles published from January 1, 2010 to June 1, 2019. The search terms used included: “health insurance,” “private or commercial health plan(s),” “private or commercial health insurance,” “private or commercial health company,” “health within six words around the word of utilization or utility,” and “hospital within six words around the word of utilization or utility” (see detailed search strategies in Additional file 1). We searched for additional references by cross-checking the reference lists of the studies retrieved and of relevant reviews. We also contacted researchers in the field to identify trials that were eligible for inclusion.

### Inclusion and exclusion criteria

We included both prospective and retrospective longitudinal controlled studies in this systematic review. Studies were eligible if they described original empirical research on the utilization of health care by individuals with private health insurance. The eligibility criteria were: 1) original studies (randomized controlled trial, case-control, cohort, cross-sectional, or pre-post); 2) one group of study participants with private health insurance (exposure group); 3) one group of study participants without private insurance (control); 4) utilization of health care [outpatient services: emergency department (ED) visits, clinic visits; inpatient services: length of stay (LOS), hospitalization rate] as an outcome [9]; 5) publication in the English language in 2010 or later. Reviews, commentaries, protocols, editorials, case reports, qualitative research, and letters were excluded. Studies on diagnostic support (e.g., radiology, clinical pathology) were also excluded. If two articles were found to derive from the same study, only the original study was included. However, if different target outcomes were reported, then both papers were included.

### Study selection

Titles and abstracts were first screened for relevance by two independent reviewers, and full-text articles with potential eligibility were downloaded for further assessment. When consensus could not be reached, disagreements were resolved by consulting a third author.

### Data collection

Data were collected with an extraction form validated in pilot studies. The data items extracted in this review were as follows: (1) The surname of the first author with the year in which the paper was published; (2) study design; (3) country in which the study was conducted; (4) full report or abstract; (5) target population; (6) target exposure group; (7) target control group; (8) target outcomes [emergency department (ED) visits, clinic visits, length of stay (LOS), and/or hospitalization rate]; (9) the numerical data included the number of visits to the ED, the percentages of visits to the ED, the rates of hospitalization, the rates of outpatient office visits, and the length of inpatient stays (days).

### Quality assessment

Risk of bias was assessed independently by two reviewers. We applied the ROBINS I tool to assess the risk of bias among non-randomized intervention studies [10]. Risk of bias was assessed at the study level, and these results were used to inform a GRADE evidence assessment [11].

### Statistical analysis

We performed meta-analyses of the studies to obtain a pooled estimate for the utilization of health care by individuals with private health insurance, compared with individuals without private health insurance. Odds ratios [with 95% confidence intervals (CIs)] were obtained for the rates of visits to the ED, the percentages of ED visits, and the rates of hospitalization with Review Manager 5.3 software [12]. Using the same software program, mean differences were obtained for the rates of outpatient office visits and the length of inpatient stay (days). *P*-values < 0.05 were considered as statistically significant. Between-study heterogeneity was measured using Cochrane’s Q-test and the Higgins *I*^*2*^ statistic (*P* < 0.10 or *I*^*2*^ > 50%) was considered as statistically significant heterogeneity [13]. When heterogeneity was present, a random-effect model (Der Simonian and Laird method) was applied. The fixed-effect model was used in the absence of between-study heterogeneity (*P* > 0.10 or *I*^*2*^ < 50%). As sensitivity analysis to confirm the robustness of our results, we performed a subgroup analysis for the control arm of no private health insurance in order to distinguish individuals with no insurance from individuals with public insurance.

## Results

### Study selection

**Fig. 1:**
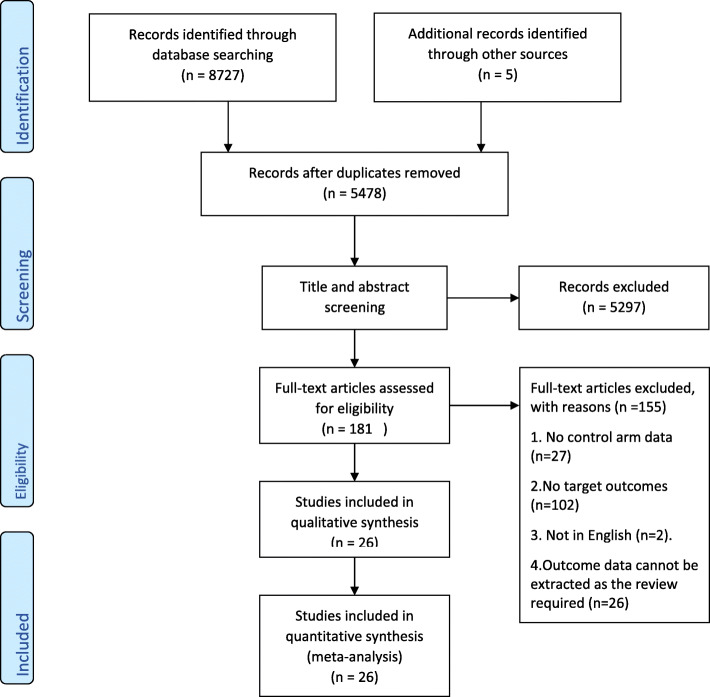
PRISMA flow diagram detailing the search strategy and results

A total of 8727 articles were selected by searching the selected electronic databases, and an additional five records were identified by cross-checking the reference lists of retrieved studies or relevant reviews. After excluding duplicates and screening titles and abstracts, we obtained 181 articles for full-text review. We eliminated 155 papers from among the 181 originally identified, based on our inclusion and exclusion criteria. Ultimately, 26 articles were included in the analysis (Fig. 1).

### Study characteristics

The basic characteristics and target outcomes of included studies are listed in Table 1. All included articles (*n* = 26) were observational studies, in the form of abstract (*n* = 6) or full report (*n* = 20). The studies included in the meta-analysis had been conducted in the United States (57.7%, 15/26), Brazil (11.5%, 3/26), South Korea (7.7%, 2/26), Australia (7.7%, 2/26), India (7.7%, 2/26), Japan (3.8%, 1/26), and Germany (3.8%, 1/26). The study populations ranged from healthy controls to patients with specific diseases or medical conditions. Among the 26 studies included, 13 (50%) focusing on the comparison between private insurance and both no insurance and public insurance, 8 (30.8%) on the comparison between private insurance and no public insurance, and 5 studies (19.2%) compared private insurance with a lack of insurance.

**Table 1:** Characteristics of the included articles

Author, year of publication	Full Report or Abstract	Country	Study Design	Study Population	Target Exposure Group	Target Control Group (s)	Target Outcome(s)
Abougergi et al. [14] 2019	Full report	The United States	Retrospective cohort study	Patients with nonvariceal upper gastrointestinal hemorrhage	Private insurance	Nondisabled public insurance	Inpatient service (LOS)
Abraham et al. [15] 2014	Full report	The United States	Retrospective cohort study	The Affordable Care Act (ACA) target population	Private insurance	Public insurance and no insurance	Outpatient service (ED visit and outpatient visit) and inpatient service (rate of hospitalization)
Abraham et al. [16] 2017	Abstract	India	Retrospective cohort study	Patients with breast, oral and ovarian cancer	Private insurance	No insurance and two kinds of public insurance	Outpatient service (outpatient visits)
Abraham et al. [17] 2017	Abstract	India	Retrospective cohort study	Patients with breast cancer	Private insurance	No insurance and public insurance	Inpatient service (LOS)
Araujo et al. [18] 2017	Full report	Brazil	Retrospective case control study	Adults ≥18 years of age.	Private health insurance	No private insurance	Outpatient service (outpatient visits) and inpatient services (rate of hospitalization)
Bhandari et al. [19] 2018	Abstract	The United States	Retrospective cohort study	Adults aged 18–64 years	Commercial insurance (qualified health plans, QHPs)	Public insurance (Medicaid)	Outpatient service (ED visit and outpatient visit) and inpatient service (rate of hospitalization)
Cunningham et al. [20] 2018	Full text	The United States	Retrospective cohort study	Population of California counties	Private insurance	No insurance and public insurance	Outpatient service (outpatient visits) and inpatient services (rate of hospitalization)
Dabbous et al. [21] 2014	Abstract	The United States	Retrospective cohort study	Adult diabetic patients	Private insurance	No insurance and public insurance	Outpatient service (outpatient visits)
Fontenelle et al. [22] 2018	Full report	Brazil	Retrospective cohort study	Household survey population	Private health insurance	No insurance coverage	Outpatient service (outpatient visits)
Gandhi et al. [23] 2014	Full report	The United States	Retrospective case control study	Patients with non-emergency visits	Private health insurance	No insurance and public insurance (Medicare and Medicaid)	Outpatient service (outpatient visits)
Ginde et al. [24] 2012	Full report	The United States	Cross-sectional household interview survey	noninstitutionalized US civilian population	Private insurance	Public insurance (Medicare and Medicaid)	Outpatient service (ED visit)
Halpern et al. [25] 2011	Full report	The United States	Retrospective cohort study	Individuals with epilepsy	Private insurance	No insurance and public insurance (Medicare and Medicaid)	Outpatient service (ED visit and outpatient visit) and inpatient service (inpatient LOS and rate of hospitalization)
Hasegawa et al. [26] 2014	Full report	The United States	Retrospective case control study	Patients ages 18 to 54 years with acute asthma	Private health insurance	No health insurance and public health insurance	Outpatient service (ED visit)
Henke et al. [27] 2013	Full report	The United States	Retrospective cohort study	Inpatient patients	Private health insurance	Public insurance (Medicare)	Inpatient service (LOS)
Hullegie et al. [28] 2010	Full report	Germany	Retrospective cohort study	West German individuals	Private insurance	Public insurance	Outpatient service (outpatient visit) and inpatient service (inpatient LOS)
Jeon et al. [29] 2013	Full report	South Korea	Retrospective cohort study	Adults participating in Korea Health Panel Survey (KHPS)	Private insurance	No private insurance	Outpatient service (outpatient visit) and inpatient service (inpatient LOS and rate of hospitalization)
Leach et al. [30] 2012	Full report	Australia	Retrospective cohort study	Participants aged 15–93 years old	Private health insurance	No insurance	Outpatient service (outpatient visit)
Mandsager et al. [31] 2015	Full report	The United States	Retrospective cohort study	Health center patients	Private insurance	Public insurance	Outpatient service (outpatient visit)
Pomerantz et al. [32] 2013	Abstract	Brazil	Retrospective cohort study	Survey adults in Brazil	Private insurance	Public insurance	Outpatient service (ED visits) and inpatient service (inpatient LOS and rate of hospitalization)
Rice et al. [33] 2014	Full report	The United States	Retrospective cohort study	Diabetic Patients	private/employer-sponsored insurance (ESI)	Public insurance (Medicaid)	Outpatient service (ED visits) and inpatient service (inpatient LOS)
Sarkar et al. [34] 2017	Full report	The United States	Retrospective cohort study	Population of children with special health care needs (CSHCN) in Ohio	Private insurance	Public insurance (Medicaid)	Outpatient service (ED visits) and inpatient service (rate of hospitalization)
Shmueli et al. [35] 2014	Full report	Australia	Retrospective cohort study	Inpatients of the public New South Wales hospitals	Private insurance	Public insurance	Inpatient service (inpatient LOS)
Terveen et al. [36] 2015	Abstract	The United States	Retrospective cohort study	Pediatric ophthalmic inpatients	Private insurance	Public insurance (Medicaid)	Inpatient service (inpatient LOS)
Yoshioka et al. [37] 2010	Full report	Japan	Retrospective cohort study	Community-dwelling frail elderly people	Private insurance provided by private care management agencies	Public insurance provided by social welfare corporations or public agencies)	Outpatient service (outpatient visit)
You et al. [38] 2018	Full report	South Korea	Retrospective cohort study	Diabetes outpatients	Supplementary private health insurance (SPHI)	Without SPHI	Outpatient service (outpatient visits) and inpatient services (rate of hospitalization)
Young et al. [39] 2009	Full report	The United States	Retrospective cohort study	Children with autism	Private insurance	Public insurance (Medicaid)	Outpatient service (outpatient visits)

*Abbreviations*: *LOS* length of stay, *ED* emergency department

### Risk of bias

**Fig. 2:**
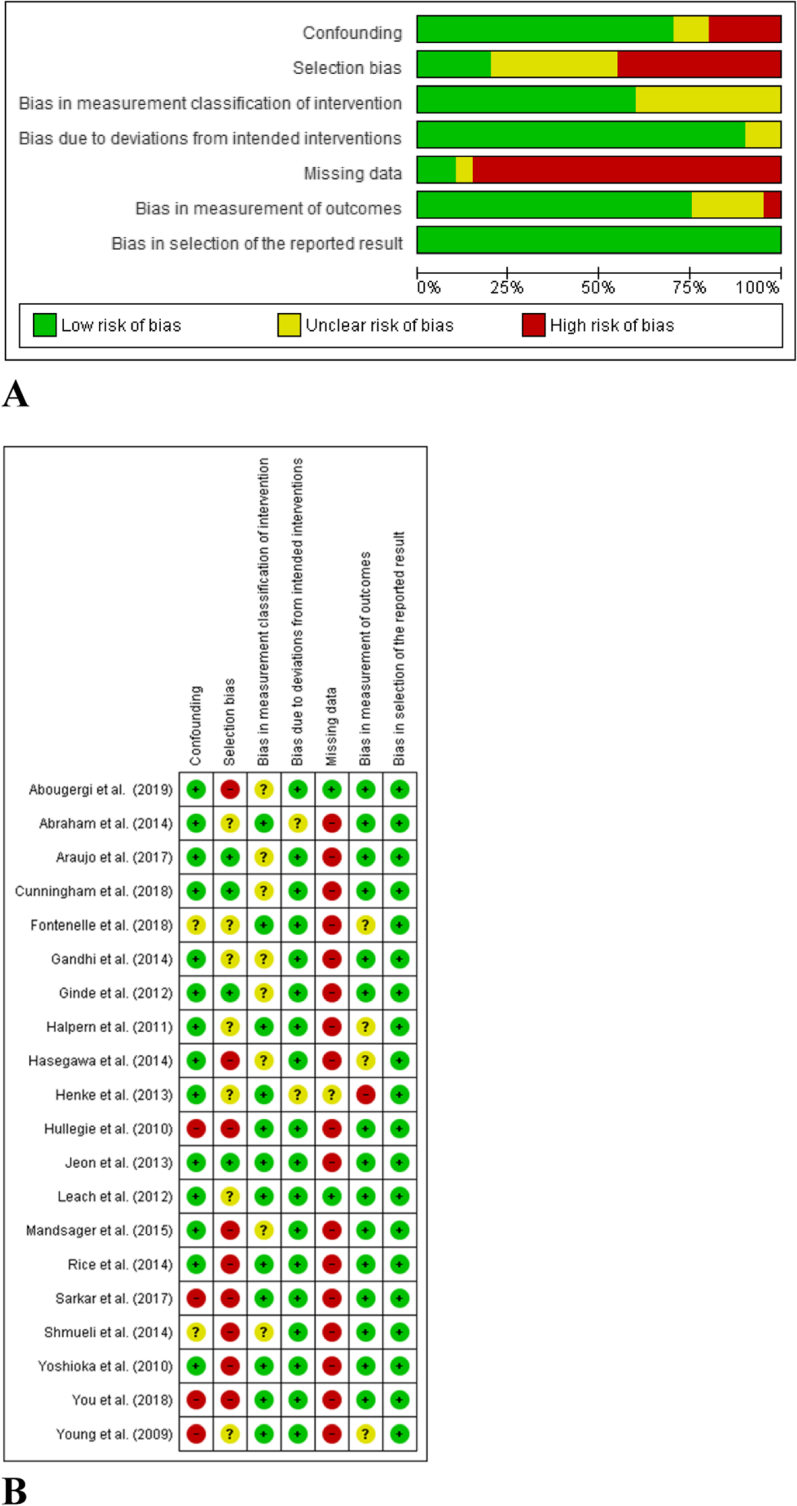
Risk of bias assessment. **a** Risk of bias graph: review authors’ judgements about each risk of bias item presented as percentages across all included full reported studies (*n* = 20). **b** Risk of bias summary: review authors’ judgements about each risk of bias item for each included full reported study

We evaluated risk of bias for all full reports included in the meta-analysis (*n* = 20) with the ROBINS I tool. We did not assess the risk of bias in abstracts because there was insufficient information for the evaluation of methodological quality. Figure 2a shows the risk of bias for each cohort. Evaluations for each domain are shown in Fig. 2b. These figures did not include studies reported as abstracts only.

### Utilization of outpatient services

All detailed data extraction results can be found in Additional file 1.

#### ED visits

**Fig. 3:**
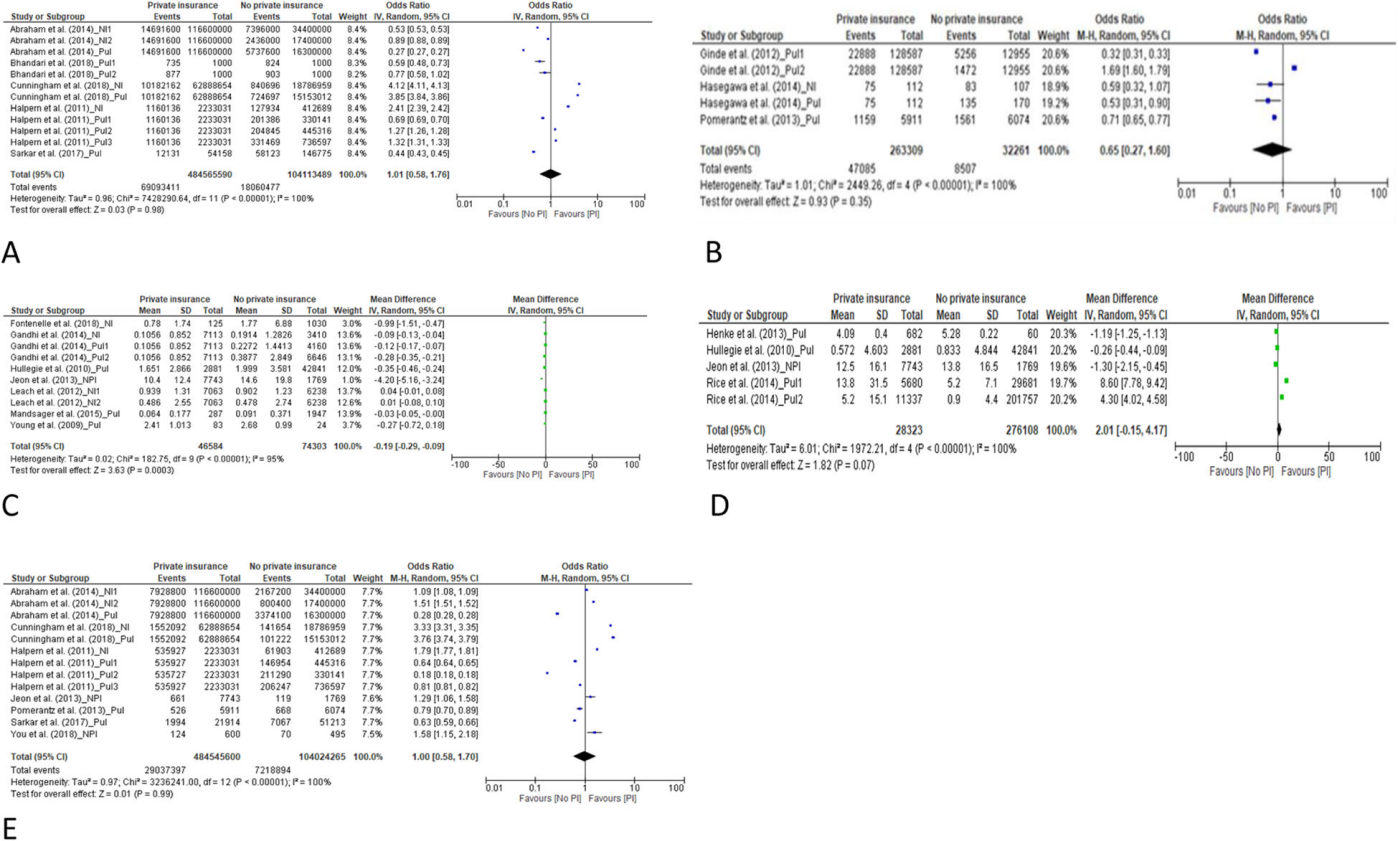
Forest plots of the total pooling results. **a** for comparison of the rates of emergency department visits between private insurance and no private insurance. **b** for comparison of the percentages of emergency department visits between those with private insurance and those without private insurance. **c** for comparison of the rates of outpatient office visits between private insurance and no private insurance. **d** for comparison of the rates of outpatient office visits between private insurance and no private insurance. **e** for comparison of the length of inpatient stay (days) between those with private insurance and those without private insurance

**Fig. 4:**
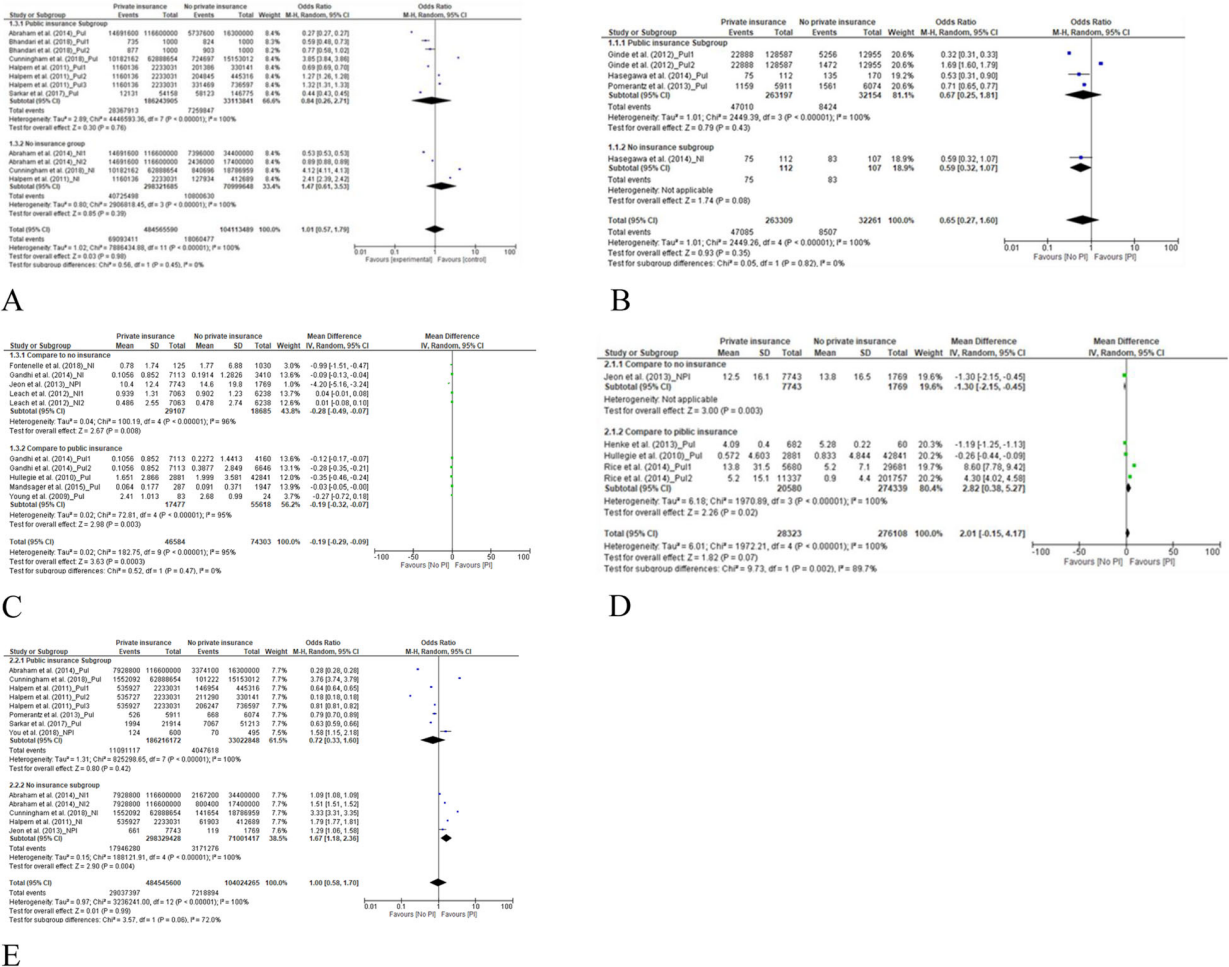
Forest plots of subgroup analysis according to the control of public insurance and no health insurance. **a** for comparison of the rates of emergency department visits between private insurance and no private insurance. **b** for comparison of the percentages of emergency department visits between those with private insurance and those without private insurance. **c** for comparison of the rates of outpatient office visits between private insurance and no private insurance. **d** Comparison of the length of inpatient stay (days) between those with private insurance and those without private insurance. **e** Comparison of the rates of hospitalization between individuals with private insurance and those with no private insurance

We used the data from 5 studies, which collectively included > 500,000,000 participants, to determine the odds ratio (OR) for a comparison of the rates of ED visits among people with private insurance, compared to people without private insurance [15, 19, 20, 25, 34]. The pooled results yielded an OR of 1.01 (95% CI 0.58–1.76) (Fig. 3a). There was no significant difference between people with private insurance and people without private insurance in the rate of ED visits. The results of subgroup analysis showed that this OR was similar for people with public insurance and people with no insurance (Fig. 4a).

Three included studies, which included 285,570 participants, reported the percentage of study participants who had visited the ED [24, 26, 32]. The proportion of those with private insurance who visited the ED was similar to the proportion of people without private insurance who visited the ED. The OR for pooled results was 0.65 (95% CI 0.27–1.60). See Fig. 3b.

The results of subgroup analysis (Fig. 4b) showed that there was no significant difference between the percentage of people with private insurance who visited the ED and either those with public insurance or those with no insurance at all.

#### Rate of outpatient office visits

We used the data from 7 studies, which included 120,887 participants, to determine the mean difference in the rate of outpatient office visits between people with private insurance and people without private insurance. After pooling the results, the mean difference was − 0.19 (95% CI − 0.29 to − 0.09) (see forest plot in Fig. 3c). People with private insurance were significantly less likely to visit the hospital as outpatients than people without private insurance. According to the subgroup analysis, people with private insurance were less likely to visit the outpatient office, compared to people with public insurance, and also compared to people without insurance (*P* < 0.05) (see forest plot in Fig. 4c). In Additional file 1, we present the data pertaining to outpatient visits that could not be included in the meta-analysis (9 articles) [15–18, 21, 25, 32, 37, 38]. The favorable results (more outpatient visits) for both people with private insurance and people without private insurance were reported.

### Utilization of inpatient services

#### Inpatient LOS

We used the data from 4 studies, which included 304,431 participants, to determine the mean difference in LOS (days) between people with private insurance and people without private insurance [27–29, 33]. The mean difference in pooled results was 2.01 (95% CI − 0.15 to 4.17, Fig. 3d). There was no significant difference between people with private insurance and people without private insurance in terms of inpatient LOS.

According to the subgroup analysis (Fig. 4d), compared to people with public insurance, people with private insurance were more likely to stay longer in the hospital (mean difference (days) = 2.82, 95% CI 0.38–5.27). While there was only one study left for compared to people without private insurance with the results of mean difference of LOS (− 1.30, 95% CI − 2.15 to − 0.45), which means the favorite result (longer of LOS) for people without private insurance.

In Additional file 1, we list the data for the mean difference in LOS from 6 articles that could not be included in the meta-analysis [14, 16, 19, 25, 35, 36]. Favorable results (longer LOS) for both people with private insurance and people without private insurance were reported.

#### Rate of hospitalization

We used the data from 7 studies, which included > 500,000,000 participants in determining the OR for the rate of hospitalization among those with private insurance, compared with those without private insurance [15, 20, 25, 29, 32, 34, 38]. The OR for the pooled results was 1.00 (95% CI 0.58–1.70) (see forest plot in Fig. 3e). There was no significant difference in the rate of hospitalization between people with private insurance and people without private insurance.

According to the subgroup analysis (Fig. 4e), those with public insurance and those with private insurance had similar rates of hospitalization (OR = 0.72, 95% CI 0.33–1.60). Compared to people with no insurance, people with private insurance were more likely to be hospitalized (OR 1.67; 95% CI, 1.18–2.36).

### The certainty of the evidence (GRADE)

The certainty of the evidence ranged from low to moderate. The observational study design meant the GRADE rating started as moderate certainty (Table 2), and almost all studies (except Abougergi et al. 2019) were missing data. Furthermore, we considered it likely that possible biases and confounding factors would have had a significant impact on the results presented in abstract form only.

**Table 2:** GRADE evidence profile: Healthcare service utilization for people with private insurance and without private insurance

Certainty assessment							№ of patients		Effect		Certainty	Importance
№ of studies	Study design	Risk of bias	Inconsistency	Indirectness	Imprecision	Other considerations	[PI]	[NPI]	Relative (95% CI)	Absolute (95% CI)		
**Percentage of ED visits**												
5	observational studies	serious ^a^	serious ^b^	not serious	not serious	none	47,085/263309 (17.9%)	8507/32261 (26.4%)	**OR 0.65** (0.27 to 1.60)	**75 fewer per 1000** (from 176 fewer to 101 more)	⨁⨁◯◯ LOW	IMPORTANT
**Rate of ED visits**												
12	observational studies	serious ^c^	serious ^b^	not serious	not serious	none	69,093,411/484565590 (14.3%)	18,060,477/104113489 (17.3%)	**OR 1.01** (0.58 to 1.76)	**1 more per 1000** (from 65 fewer to 96 more)	⨁⨁◯◯ LOW	IMPORTANT
**Rate of outpatient visits**												
10	observational studies	serious ^c^	not serious	not serious	not serious	none	46,584	74,303	–	MD **0.19 lower** (0.29 lower to 0.09 lower)	⨁⨁⨁◯ MODERATE	IMPORTANT
**Inpatient length of say**												
5	observational studies	serious ^a^	serious ^b^	not serious	not serious	none	28,323	276,108	–	MD **2.01 higher** (0.15 lower to 4.17 higher)	⨁⨁◯◯ LOW	IMPORTANT
**Rates of hospitalization**												
13	observational studies	serious ^c^	serious ^b^	not serious	not serious	none	29,037,397/484545600 (6.0%)	7,218,894/104024265 (6.9%)	**OR 1.00** (0.58 to 1.70)	**0 fewer per 1000** (from 28 fewer to 43 more)	⨁⨁◯◯ LOW	IMPORTANT

*CI* Confidence interval, *OR* Odds ratio, *MD* Mean difference, *PI* private insurance, *NPI* no private insurance

Explanations

^a^ All included full reports had the problem of missing data

^b^ Favorable results for both people with private insurance and people without private insurance were reported

^c^ Having the problem of missing data and some data came from abstracts

## Discussion

In this systematic review, we investigated whether people with private insurance were more likely to utilize health care than those without private insurance. According to the results of the meta-analysis, the utilization was similar between those with and those without private health insurance. For the target outcome of outpatient office visits, people with private insurance were less likely to visit the outpatient office than people without private insurance (mean difference = − 0.19 (95% CI − 0.29 to − 0.09)). In theory, people with private insurance should have more access to health care. However, our results indicate that there was no significant increase in the consumption of healthcare services among individuals with private health insurance. In one of the dimensions examined, those with private health insurance coverage actually used fewer of the health care available to them. One possible explanation is that the utilization of medical services was more directly correlated with the need for the service than with insurance coverage, as suggested by previous studies [40–42]. Private health insurance coverage does not appear to increase the utilization of health care and may ease the financial burdens on patients and social health insurance plans.

The results of subgroup analysis to identify differences between those without insurance and those with public insurance showed that most results were consistent with the total pooled results. For LOS, people with private insurance were more likely to stay longer in the hospital, compared to people with public insurance (mean difference (days) = 2.82, 95% CI 0.38–5.27). With regard to the rate of hospitalization, compared to people without any insurance, people with private insurance were more likely to be hospitalized (OR 1.67; 95% CI, 1.18–2.36). As inpatient services are more tightly linked to medical necessity than outpatient services, these results reflect the potential for private insurance to relieve patients’ financial burden.

To our best knowledge, this systematic review is the first review to assess the impact of private insurance coverage on the utilization of health care across the globe. This study strictly followed the standards for systematic reviews, including explicit eligibility criteria, duplicated independent assessments of eligibility, and a comprehensive literature search. One limitation of this study was that more than half of the included studies were conducted in the United States, which restricted the external validity of the results. Another limitation of this review is that the results may have been confounded by selection bias due to divergences in methodology among health care systems. Next, the evidence of this present study has temporal limitations. Studies on this topic were conducted prior to 2010. However, we restricted the search period to years from 2010 onward in order to focus our investigation on current insurance policy. Finally, as there is no standardized tool for the assessment of abstract quality, all abstracts included in the review were not graded in terms of quality. This fact may limit the ability of other researchers to extrapolate from the results reported here. Additional studies will be necessary to explore these issues.

## Conclusion

People with private insurance did not increase their utilization of health care (outpatient services), compared to those without private insurance. Private health insurance coverage may ease the financial burdens on patients and on the public health insurance system.

## Supplementary information

**Additional file 1.**

## Data Availability

The datasets used during the current study are available from the corresponding author on reasonable request.

## Abbreviations

PRISMA: Preferred Reporting Items for Systematic Reviews and Meta-Analyses
CENTRAL: Cochrane Central Register of Controlled Trials
ED: Emergency department
LOS: Length of stay
CIs: Confidence intervals
OR: Odds ratio
