# Supplementary material for: Utilization of public health care by people with private health insurance: a systematic review and meta-analysis
Source: BMC Public Health. 2020 Jul 23;20:1153. doi: 10.1186/s12889-020-08861-9 (PMC7376853; doi:10.1186/s12889-020-08861-9)
Supplement: Supplementary file 1 — Additional file 1. [file 12889_2020_8861_MOESM1_ESM.docx]

**Appendix I. Search strategies.**

***1. Database: OVID Medline Epub Ahead of Print, In-Process & Other Non-Indexed Citations, Ovid MEDLINE(R) Daily and Ovid MEDLINE(R) 1946 to Present***

Search Strategy:

--------------------------------------------------------------------------------

1 ((health insurance or private health plan*or commercial health plan* or private health insurance or commercial health insurance or private health company* or private insurance or commercial insurance) and ((utili* adj6 health*) or (utili* adj6 hospital*))).mp. [mp=title, abstract, original title, name of substance word, subject heading word, floating sub-heading word, keyword heading word, organism supplementary concept word, protocol supplementary concept word, rare disease supplementary concept word, unique identifier, synonyms] (2855)

2 limit 1 to (humans and English and yr="2009 -Current") (1499)

***2. Database: Embase <1996 to 2019 June 04>***

Search Strategy:

--------------------------------------------------------------------------------

1 ((health insurance or private health plan*or commercial health plan* or private health insurance or commercial health insurance or private health company* or private insurance or commercial insurance) and ((utili* adj6 health*) or (utili* adj6 hospital*))).mp. [mp=title, abstract, heading word, drug trade name, original title, device manufacturer, drug manufacturer, device trade name, keyword, floating subheading word, candidate term word] (10095)

2 limit 1 to (humans and English and yr="2009 -Current") (6630)

***3. Database: Cochrane Library***

Last Saved: 05/06/2019

Comment:

ID Search

#1 (health insurance or private health plan*or commercial health plan* or private health insurance or commercial health insurance or private health company* or private insurance or commercial insurance): ti, ab, kw (3473)

#2 Utili* (51134)

#3 Health* (368583)

#4 Hospital* (320544)

#5 #2 AND #3 (27239

#6 #2 AND #4 (18744)

#7 #5 OR #6 (33360)

#8 #8 AND #1 (616) (16 reviews, 2 protocols, and 598 trials)

**Appendix II.** Data extraction results from the included articles.

| Study (year) | Outpatient service | | Inpatient service | |
| --- | --- | --- | --- | --- |
|  | ED visits (per patient) | Outpatient visits (per patient) | LOS (mean days per year ±SD) | Hospitalization |
| Abougergi *et al.* (2019) |  |  | PI vs. PuI = mean adjusted difference: −0.26 d (95%CI: −0.40 to −0.13 d), P< 0.01. |  |
| Abraham *et al.* (2014) | Rate of ED visits per year:  NI=0.215 (n=34.4 million);  NI=0.140 (17.4 million);  PI=0 .126 (n= 116.6 million); PuI=0.352 (n= 16.3 million) | Rate of outpatient visits per year: NI=2.14 ((n=34.4 million)  or 2.91(17.4 million); PI=5.25 (n= 116.6 million); PuI =8.32 (n= 16.3 million) |  | Average number of inpatient stays: NI=0.063 (n=34.4 million) or 0.046 (17.4 million), PI=0.068 (n=116.6 million), PuI=0.207 (n=16.3 million) |
| Abraham et al. (2017) |  | Rate of outpatient visits per year:  for BC: NI=11.8; PI=16.3; PuI_1_=2.7  PuI_2_ =2.2  For OC, NI= 24.6; PI=36.5; PuI_1_=8;  PuI2=1.2  For OVC, NI=35.9; PI=30.3; PuI1=19.5; PuI2 =1.1 |  |  |
| Abraham *et al.* (2017) |  |  | NI=2.6 ±1.8  PI =4.3±3.5  PuI = 3.5±2.28 |  |
| Araujo *et al.* (2017) |  | PI: NPI (Adjust prevalence ratio(95%CI)) = 1.14 (1.10 to 1.19) |  | PI: NPI (Adjust prevalence ratio (95%CI)) = 1.07 (0.78 to 1.47) |
| Bhandari *et al.* (2018) | Rate of ED visits per 100 patients, Year 1:  PI = 73.5±1.0, PuI= 82.4±1.2;  Year 2: PI= 87.7±1.7, PuI = 90.3±1.9. |  | Year 1: PI = 6.2±0.2, PuI=4.7±0.2;  Year 2: PI=6.2±0.4, PuI =4.4±0.2. | PI = 643.0±22.2;  PuI =757.7±23.0 (rates of per 10000 patients) |
| Cunningham *et al.* (2018) | Rate of ED visits per year:  NI= 4.47% (n= 18786959)  PI=16.2% (n= 62888654)  PuI=4.78% (n= 15153012) |  |  | Rate of inpatient admission per year:  NI= 0.754% (n= 18786959)  PI=2.468% (n= 62888654)  PuI=0.668% (n= 15153012) |
| Dabbous *et al.* (2014) |  | Rate of outpatient visits per year:  NI: PI: PuI  =0.4:0.9:1 |  |  |
| Fontenelle *et al.* (2018) |  | Rate of outpatient visits per year:  PI (mean ±SD) = 0.78 ± 1.74, (n=125);  NI (mean ±SD) = 1.77 ± 6.88, (n=1030) |  |  |
| Gandhi *et al.* (2014) |  | 2009 Adjusted Nonemergent Visit Rates per patient: PI=0.1056 ±0.852(n=7113), NI=0.1914 ±1.2826 (n=3410), PuI=0.2272 ±1.4413 (n=4160) or 0.3877 ±2.849 (n=6646) |  |  |
| Ginde *et al.* (2012) | Percentage of ED visit per year:  PI=17.8% (n=128587)  PuI= 39.8 (n=12955)  or 26.8 (n=12955) |  |  |  |
| Halpern *et al.* (2011) | Rate of ED visits per year:  PI=0.52 (n=2,233,031), PuI= 0.46 (n=445,316),  or  0.61(n=330,141) or 0.45(n=736,597), NI= 0.31 (n=412,689) | Rate of outpatient visits per year:  PI= 10.31, PuI=17.8 or 10.76 or 9.69  NI= 3.36 | PI=7.91, PuI= 8.64, 14.66 or 7.44, NI= 2.92 (days) | Average annual rates: PI= 0.24, PuI= 0.33, 0.64 or 0.28, NI=0.15 |
| Hasegawa *et al.* (2014) | Percentage of ED visit per year:  PI=67.0% (n=112); PuI=79.4% (n=170); NI=77.5% (n=107) |  |  |  |
| Henke *et al.* (2013) |  |  | PI=4.09±0.4 (n=682), PuI =5.28±0.66 (n=60). |  |
| Hullegie *et al.* (2010) |  | Rate of outpatient visits per year:  PuI= 1.999  ±3.581 (n= 42 841), PI= 1.651  ±2.866 (n= 2881) | PuI=0.833  ±4.844, PI= 0.572  ±4.603 |  |
| Jeon *et al.* (2013) |  | Rate of outpatient visits per year:  PI= 10.4 ± 12.4 (n=7743), NPI= 14.6 ± 19.8 (n=1769) | PI= 12.5 ± 16.1, NPI= 13.8 ± 16.5 | PI=8.5% (n=661/7743);  NPI=6.7% (n=119/1769) |
| Leach *et al.* (2012) |  | Rate of outpatient visits per year:  PI=0.939 ± 1.31 (n=7063), NI= 0.902 ±1.23 (n=6238) for GP; PI= 0.486 ±2.55 (n=7063), NI= 0.478 ±2.74 (n=6238) for specialists. |  |  |
| Mandsager *et al.* (2015) |  | Rate of outpatient visits per year: PI=0.064±0.177(n=287), PuI= 0.091 ±0.371(n=1947) |  |  |
| Pomerantz *et al.* (2013) | Percentage of ED visit per year:  PI=19.6% (n=5,911), PuI=25.7% (n=6,074). | Percentage of outpatient visits per year:  PI=70.9% (N=5,911), PuI=86.0% (N=6,074). |  | PI=8.9% (N=5,911), PuI=11.0% (N=6,074). |
| Rice *et al.* (2014) | Rate of ED visits per year:  PI= 1.3 ±3.9 (n=5680) vs. PuI = 1.0 ±1.7 (n=29681)  or PI= 0.5±1.4 (n=113337) vs. PuI= 0.6 ±1.3 (n=201757) |  | PI= 13.8 ±31.5 or 5.2 ±15.1, PuI = 2.0 ±7.1 or 0.9 ± 4.4 |  |
| Sarkar *et al.* (2017) | Rate of ED visits per year:  PuI=39.6%±2.42% (n= 146 775), PI=22.4%± 2.17% (n= 54 158). |  |  | PuI=13.8%±1.69% (N= 51 213), PI=9.1%± 1.45% (N= 21 914). |
| Shmueli *et al.* (2014) |  |  | PI=5.510 (N=13,516), PuI= 5.457 (N= 79,548) |  |
| Terveen *et al.* (2015) |  |  | PI= 3.11 days, PuI= 3.43 days (P<0.001) |  |
| Yoshioka et al. (2010) |  | Rate of outpatient visits per year:  PuI= 61, PI= 33 |  |  |
| You et al. (2018) |  | Percentage of outpatient visits per year:  PI= 79.3% (n=2304), NPI=85.8% (n=2980) |  | PI= 20.7% (n=600), NPI=14.2% (n=495) |
| Young et al. (2009) |  | Rate of outpatient visits per year:  PI= 2.41 ±1.013 (n=83), PuI= 2.68 ±0.990 (n=24) |  |  |

**Abbreviation:** NI for no insurance; PI for private insurance; PuI for public insurance; NPI for no private insurance; BC for breast cancer; OC for oral cancer; OVC for ovarian cancer; NPI for not private insurance; SD standard deviation; CI: 95% confidence interval; LOS for length of stay; GP general practitioner

**Appendix III.** The comparison of outpatient visits rates between private insurance, no insurance, and public insurance with the data that cannot perform meta-analysis in the included articles.

| **Study ID** | **Outpatient visits rates (mean rates per patient otherwise noted)**  **No insurance (NI) vs. private insurance (PI)** | **Outpatient visits rates (mean rates per patient otherwise noted)**  **Public insurance (PuI) vs. PI** |
| --- | --- | --- |
| Abraham et al. (2014) | *NI vs. PI=2.14:5.25 (p<0.05)  *NI vs. PI=2.91:5.25 (P<0.05) | PuI vs. PI=8.32: 5.25 (no *P* value) |
| Abraham et al. (2017)[23] | *NI vs. PI=11.8 :16.3 (p< 0.001) | *PuI_1_ vs. PI_1_=2.7 :16.3 (p< 0.001)  *PuI_2_ vs. PI_1_=2.2 :16.3 (p< 0.001) |
| Abraham et al. (2017)[22] | *NI vs. PI=24.6: 36.5 (p< 0.001)  ^NI vs. PI=35.9 :30.3 (p< 0.001) | *PuI_1_ vs. PI_2_=8 :36.5 (p< 0.001)  *PuI_2_ vs. PI_2_=1.2 :36.5 (p< 0.001)  PuI_3_ vs. PI_3_=19.5 :30.3 (p< 0.001)  PuI_4_vs. PI_3_ =1.1 :30.3 (p< 0.001) |
| Araujo et al. (2017) | *PI: NI (Adjust prevalence ratio(95%CI)) =1.14 (1.10 to 1.19) |  |
| Dabbous et al. (2014) | NI vs. PI = 0.4:0.9 (no *P* value) | PI vs. PuI =0.9:1 (no *P* value) |
| Halpern et al. (2011) | *NI vs. PI==3.36: 10.31 (*P* <0.05) | ^PuI_1_ =17.8 vs. PI=10.31 (*P* <0.05)  PuI_2_=10.76 vs. PI=10.31 (*P*>0.05)  PuI_3_ =9.69 vs. PI=10.31 (*P*>0.05) |
| Pomerantz et al. (2013) |  | PuI =70.9% vs. PI=86.0% (physician consultations) (*P* <0.05) |
| Yoshioka et al. (2010) |  | PuI =61 vs. PI=33 (use rates of medical service) |
| You et al. (2018) | ^NPI vs. PI==56.4%: 43.6% (p<0.001) |  |

*show the favorable results (more outpatient visits) of people with private insurance with statistic significance (p<0.05).

**^** show the favorable results of people without private insurance with statistic significance (p<0.05).

**Appendix IV.** The comparison of inpatient length of stay between private insurance, no insurance, and public insurance with the data that cannot perform meta-analysis in the included articles.

| **Study ID** | **LOS (days)**  **No insurance (NI) vs. private insurance (PI)** | **LOS (days)**  **Public insurance (PuI) vs. private insurance (PI)** |
| --- | --- | --- |
| Abougergi et al. (2019) |  | ^PI vs. PuI (mean adjusted difference): −0.26 days (95%CI, −0.40 to −0.13 d), P< 0.01. |
| Abraham et al. (2017) | *NI (mean ±SD): 2.6 ±1.8  *PI (mean ±SD): 4.3±3.5 (p< 0.001) | *PuI (mean ±SD): 3.5±2.28  *PI (mean ±SD): 4.3±3.5 (p< 0.001) |
| Bhandari et al. (2018) |  | *Year 1: PuI (mean ±SD): 4.7±0.2 vs. PI (mean ±SD): 6.2±0.2 (p< 0.001)  *Year 2: PuI (mean ±SD): 4.4±0.2 vs. PI (mean ±SD): 6.2±0.4, (p< 0.001) |
| Halpern et al. (2011) | *PI vs. NI=7.91:2.92 (p<0.05) | PuI_1_ vs. PI=8.64: 7.91 (P>0.05)  ^PuI_2_ vs. PI= 14.66:7.91 (p<0.05)  PuI_3_ vs. PI =7.44: 7.91(P>0.05) |
| Shmueli et al. (2014) |  | PuI vs. PI = 5.457 :5.510 (no *p* value) |
| Terveen et al. (2015) |  | ^PuI vs. PI = 3.43: 3.11 (P<0.001) |

Abbreviation: LOS for length of stay; PI for private insurance; PuI for public insurance; IN for no insurance; SD for standard deviation.

*show the favorable results (longer LOS) of people with private insurance with statistic significance (p<0.05).

**^** show the favorable results of people without private insurance with statistic significance (p<0.05)
